# H_2_S Sensing with SnO_2_‐Based Gas Sensors: Sulfur Poisoning Mechanism Revealed by Operando DRIFTS and DFT Calculations

**DOI:** 10.1002/anie.202504696

**Published:** 2025-04-04

**Authors:** Tingqiang Yang, Matthias Boepple, Anne Hémeryck, Antoine Jay, Sara Karwounopoulos, Udo Weimar, Nicolae Barsan

**Affiliations:** ^1^ Institute of Physical and Theoretical Chemistry (IPC) University of Tuebingen Auf der Morgenstelle 15 D‐72076 Tuebingen Germany; ^2^ Center for Light‐Matter Interaction Sensors & Analytics (LISA^+^) University of Tuebingen Auf der Morgenstelle 15 D‐72076 Tuebingen Germany; ^3^ LAAS‐CNRS Université de Toulouse CNRS Toulouse F‐31555 France; ^4^ International Research Organization for Advanced Science and Technology (IROAST) Kumamoto University Kumamoto Japan

**Keywords:** DFT, DRIFTS, H_2_S sensor, SnO_2_, Sulfur poisoning

## Abstract

Real‐time detection of toxic and flammable H_2_S remains challenging for cost‐effective semiconducting metal oxide (SMOX) sensors due to the insufficient focus on and inherently poor understanding of the sulfur‐poisoning effect. This research, focusing on SnO_2_ as a model for SMOX sensors, identifies the formation of sticky sulfite and sulfate surface species as the root cause of poisoning through the detailed analyses of results obtained from operando diffuse reflectance infrared Fourier transform spectroscopy (DRIFTS) experiments and density functional theory (DFT) calculations. The formation of the poisoning species is highly energetically favorable. Meanwhile, the decomposition of sulfite and sulfate appears unfavorable at the typical operating temperature of 300 °C and is only feasible around the literature‐reported 500 °C. The sulfur poisoning effect is also likely to occur with SO_2_ and other sulfur‐containing volatile organic compounds (VOCs). Overcoming this issue is expected to require surface additives and/or alternative SMOX materials capable of providing different reaction pathways. The significance of metal‐sulfur‐oxygen chemistry extends beyond SMOX gas sensors to desulfurization catalysts, denitration catalysts, and solid oxide fuel cells.

## Graphical abstract

The formation of sticky sulfite and sulfate surface species is identified as the root cause of the poisoning effect of H_2_S on SnO_2_‐based sensors by the compelling results and detailed analyses from operando DRIFTS experiments and density functional theory (DFT) calculations.

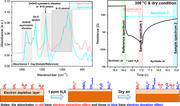

## Introduction

H_2_S is a toxic and flammable gas widely produced in oil and gas refining, naturally emitted in sewers and manure pits, and commonly used in rayon manufacturing. It causes nausea and headaches at low concentrations and can harm the respiratory and nervous systems at higher levels.^[^ ^1^ ^]^ Due to its higher density than air, it accumulates in low‐lying or enclosed spaces, posing an explosion risk. While humans can smell its strong rotten‐egg odor at part‐per‐billion levels, exposure to it can damage the olfactory nerve, making further buildup undetectable.^[^ ^2^ ^]^ Therefore, H_2_S monitoring is essential, and sensors offer an easy‐to‐deploy, cost‐effective solution.

Commercial H_2_S monitoring options include electrochemical cells, optical sensors, and semiconducting metal oxide (SMOX) sensors. SMOX sensors often excel due to their high sensitivity, low cost, compact size, and low power consumption.^[^ ^3^, ^4^, ^5^, ^6^, ^7^, ^8^, ^9^, ^10^, ^11^, ^12^, ^13^, ^14^ ^]^ Among SMOX, CuO was preferred for its well‐known semiconductor‐conductor (CuO‐CuS) transition when reacting with H_2_S.^[^ ^3^, ^4^, ^5^ ^]^ However, at low temperatures (150 °C), the transition is only partially reversible, leading to poor recovery performance. At higher temperatures (250 °C), frequent and fully reversible transitions can degrade the sensing layer's structure due to the significant lattice‐parameter difference between CuO and CuS, causing long‐term instability.^[^ ^15^ ^]^

The successfully commercialized SnO_2_ has also been widely investigated as a H_2_S sensor since Ando et al. reported the H_2_S reaction on SnO_2_ thin films in 1996.^[^ ^6^, ^7^, ^8^, ^9^, ^16^ ^]^ In 1998, Malyshev et al. proposed two possible chemical reactions of H_2_S on the SnO_2_ surface: one (Equation 1a) involves its reaction with adsorbed oxygen species (O^2−^(ads)), causing a change in resistance; the other (Equation 1b) involves its reaction with bulk SnO_2_, which may occur at high H_2_S concentrations or during prolonged exposure.^[^ ^17^ ^]^

(1a) $$H_{2}S\left(g\right)+3O^{2-}\left(\mathrm{ads}\right)\to {\mathrm{SO}}_{2}\left(g\right)+H_{2}O\left(g\right)+6e^{-}$$

(1b) $$2H_{2}S(g)\;+\;\mathrm{Sn}O_{2}\to \mathrm{Sn}S_{2}+2H_{2}O(g)$$

Based on this, three H_2_S sensing mechanisms have been proposed for different SnO_2_ nanostructures. First, in 2009, Liu et al. outlined a mechanism for a SnO_2_‐nanocrystal‐based thick‐film sensor, where H_2_S reacts with adsorbed oxygen species (O_2_^−^(ads), O^−^(ads), and O^2−^(ads), depending on temperature) to generate gas‐phase SO_2_ and H_2_ while donating electrons to the surface (Equations 2).^[^ ^6^ ^]^ The formation of SO_2_ and H_2_O was also suggested in other studies.^[^ ^18^, ^19^ ^]^

(2a) $$H_{2}S(g)+{O_{2}}^{-}\left(\mathrm{ads}\right)\to H_{2}(g)\;+\;SO_{2}\left(g\right)+e^{-}$$

(2b) $$H_{2}S\left(g\right)+2O^{-}\left(\mathrm{ads}\right)\to H_{2}\left(g\right)+{\mathrm{SO}}_{2}\left(g\right)+2e^{-}$$

(2c) $$H_{2}S\left(g\right)+2O^{2-}\left(\mathrm{ads}\right)\to H_{2}\left(g\right)+{\mathrm{SO}}_{2}\left(g\right)+4e^{-}$$

Second, in 2020, Phuoc et al. proposed a sensing mechanism for SnO_2_ porous nanofibers (Equations 3).^[^ ^7^ ^]^ At high concentrations of H_2_S, some sulfur is oxidized into SO_2_, while some is converted into SnS (Equation 3b).

(3a) $$H_{2}S(g)+3O^{-}\left(\mathrm{ads}\right)\to H_{2}O(g)\;+\;SO_{2}\left(g\right)+3e^{-}$$

(3b) $$4H_{2}S\left(g\right)\;+\;3\mathrm{Sn}O_{2}\to 4H_{2}O\left(g\right)\;+\;{\mathrm{SO}}_{2}\left(g\right)\;+\;3\mathrm{SnS}$$

Third, in 2022, Paolucci et al. proposed a mechanism involving an amorphous SnO_2_ film, where H atoms from H_2_S are captured by lattice oxygens, forming rooted hydroxyl groups (OH_rooted_) that readily ionize to release electrons. Meanwhile, sulfur bonds to two surface Sn atoms (Equations 4).^[^ ^8^ ^]^

(4a) $$H_{2}S\;\left(g\right)+2Sn_{\mathrm{Sn}}+2O_{O}↔{\mathrm{Sn}}_{\mathrm{Sn}}^{n\delta +}\text{…}S^{m\delta -}\text{…}{\mathrm{Sn}}_{\mathrm{Sn}}^{n\delta +}{+2(\mathrm{OH})}_{O}$$

(4b) $${\left(\mathrm{OH}\right)}_{O}↔{\left(\mathrm{OH}\right)}_{O}^{+}+e^{-}$$

In 2014, Lee et al. observed significantly different reaction products using ex situ X‐ray photoemission spectroscopy (XPS), demonstrating that sulfite (SO_3_^2−^) or sulfate (SO_4_^2−^) was generated at the SnO_2_ surface.^[^ ^9^ ^]^ This discovery raised concerns about the sulfur poisoning effect of H_2_S on SnO_2_. Actually, the sulfur poisoning has been extensively investigated in desulfurization catalysts, denitration catalysis, and solid oxide fuel cells.^[^ ^20^, ^21^, ^22^, ^23^, ^24^, ^25^, ^26^, ^27^, ^28^, ^29^, ^30^, ^31^, ^32^, ^33^ ^]^ Research about H_2_S photo‐oxidation and SO_2_ photo‐adsorption/photo‐fixation has shown that SO_2_ adheres strongly to metal oxide surfaces, easily transforming into sulfite, bisulfite (HSO_3_^−^), and sulfate.^[^ ^20^, ^21^, ^22^, ^23^, ^24^, ^25^, ^26^, ^27^, ^28^, ^29^, ^30^, ^31^ ^]^ The sulfur poisoning mechanisms of denitration catalysts primarily involve the formation of ammonium sulfates and metal sulfates,^[^ ^32^ ^]^ with the latter also acting as a poisoning agent for metal oxide fuel cell cathodes.^[^ ^33^ ^]^

However, the sulfur poisoning has not drawn significant attention in the gas sensor field even after the ex situ XPS research.^[^ ^9^ ^]^ The reason can be that the conditions where it was applied are very far from the application requirements. In contrast, operando diffuse reflectance infrared Fourier transform spectroscopy (DRIFTS), performed simultaneously to DC sensors resistance measurements, enables us to observe adsorbed species and reactions occurring at the surface and within the pores of the sensing layer, providing clearer and more compelling evidence of what happens under application conditions.^[^ ^34^, ^35^, ^36^ ^]^ By combining this with density functional theory (DFT) calculations, we have previously elucidated the CO reaction on SnO_2_, H_2_O splitting on WO_3_, and the HCHO sensing mechanism of In_4_Sn_3_O_12_.^[^ ^37^, ^38^, ^39^ ^]^

Herein, the H_2_S DRIFTS measurements are conducted under both dry and humid conditions to investigate the adsorption and reaction of H_2_S on the SnO_2_ surface. DFT calculations provide detailed insights into surface adsorption and reactions, focusing on active sites, reaction pathways, energy profiles, and charge effects. These findings are expected to be crucial not only for SMOX gas sensors but also for other applications involving metal‐sulfur–oxygen chemistry.

## Results and Discussions

### DRIFTS Experiments

#### Sensor Resistance

**Figure 1:**
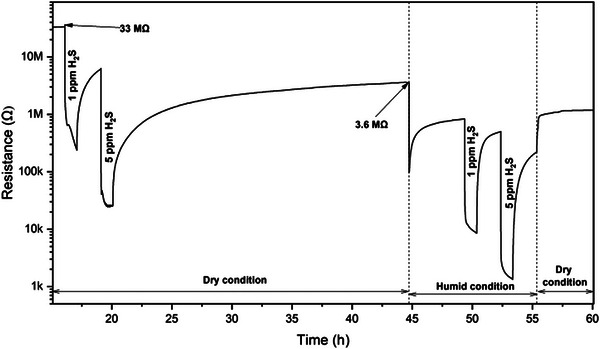
Resistance curve of SnO_2_ sensor during H_2_S DRIFTS measurement at 300 °C.

As shown in Figure 1, the SnO_2_ sensor exhibits strong responses to H_2_S. Under dry conditions, the sensor signals (the ratio of resistances in pure air to those with H_2_S) reach 141 for 1 ppm and 1281 for 5 ppm H_2_S (Table S1). Under humid conditions, the signals slightly decline but remain as high as 98 for 1 ppm and 640 for 5 ppm H_2_S. However, the SnO_2_ sensor exhibits poor recovery. The initial resistance (∼33.3 MΩ) never fully recovers during the 60‐h experiment. After exposure to 5 ppm H_2_S, under dry conditions, the baseline resistance (∼3.6 MΩ) is approximately one‐tenth of the original value, even after a 24‐h recovery period. Although recovery under humid conditions is somewhat better, it is still far from ideal.

#### DRIFTS Under Dry Conditions

**Figure 2:**
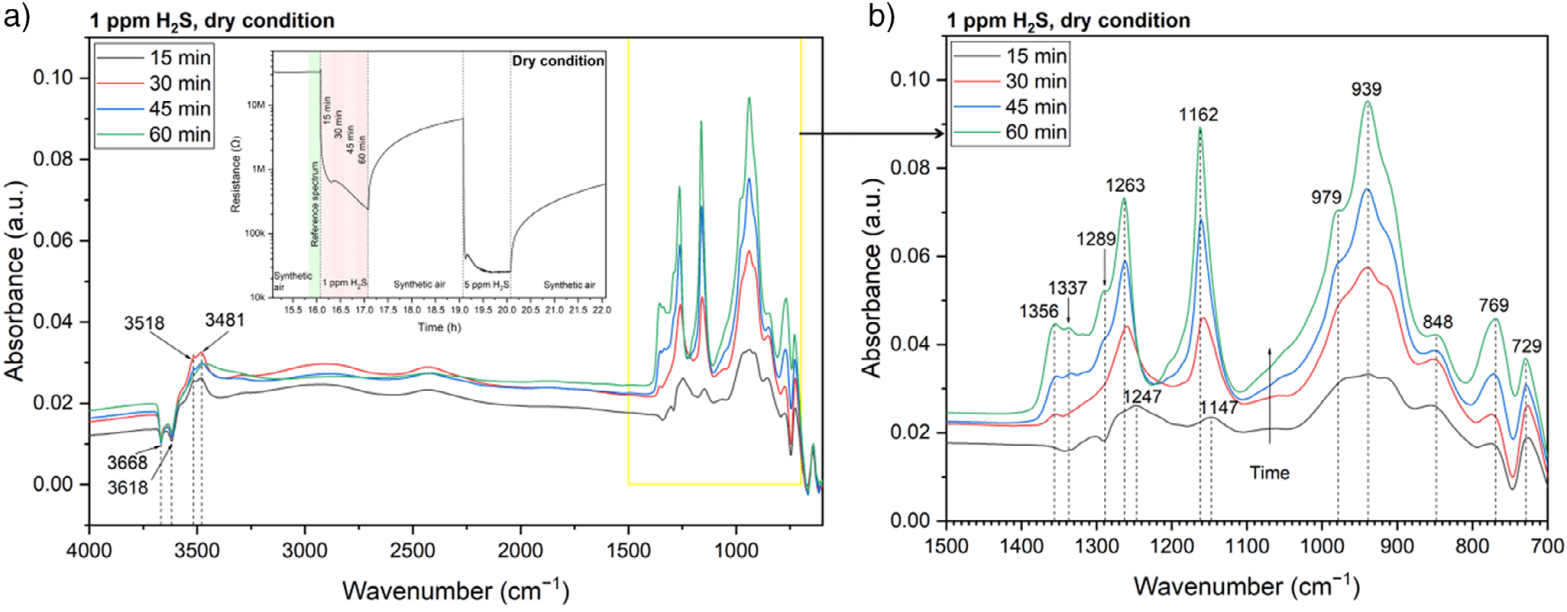
Absorbance spectra during 1‐ppm‐H_2_S exposure under dry conditions: a) the measurement range 4000–600 cm^−1^ and b) 1500–700 cm^−1^ range. The inset in panel a) shows the acquisition timings of the reference and sample spectra.

Figure 2 shows the absorbance spectra recorded during exposure to 1 ppm H_2_S under dry conditions. The details of calculating the absorbance spectra are illustrated in the Supporting Information. The absorbance spectra calculated in the same way during recovery from 1 ppm H_2_S and those for 5 ppm H_2_S are presented in Figure S3. In the absorbance spectra, increasing bands (IBs) are linked to the formation of surface groups, while decreasing bands (DBs) indicate the removal of surface groups. Significant changes are observed above 3000 cm^−1^, primarily attributed to variations in the concentration of surface hydroxyl groups, and in the 1400–800 cm^−1^ region, which pertains to previous infrared results on photo‐induced H_2_S oxidation and SO_2_ adsorption (Table 1), mainly involving sulfur‐containing species (SO_2_, SO_3_^2−^, HSO_3_^−^, SO_4_^2−^, and HSO_4_^2−^).^[^ ^20^, ^21^, ^22^, ^23^, ^24^, ^25^, ^26^, ^27^, ^28^, ^29^, ^30^, ^31^ ^]^ Thus, SO_3_^2−^/HSO_3_^−^ and SO_4_^2−^ species are likely generated on the SnO_2_ surface during and/or after the H_2_S sensing process, supporting the ex situ XPS results reported by Lee et al.^[^ ^9^ ^]^ The H─S vibration band of H_2_S around 2500 cm^−1^ is scarcely identifiable, indicating the high reactivity of H_2_S at the SnO_2_ surface. An infrared band for H_2_S was detected at 2581 cm^−1^ in research where 2500 ppm O_2_ was used to oxidize 5000 ppm H_2_S, and the band disappears at temperatures above 190 °C.^[^ ^21^ ^]^ Herein, measurements are conducted with only 1 and 5 ppm H_2_S in synthetic air (∼21% O_2_) at 300 °C. It is reasonable to assume that all H_2_S molecules are consumed immediately.

**Table 1:** Vibrational frequency of sulfur‐containing species on metal oxide surfaces as well as the hydroxyl group and Sn─O bond on SnO_2_ surface.

Species	Wavenumber/(cm^−1^)	Metal Oxides
H_2_S	2581	CeO_2_ [21]
	2560, 1335	γ‐Al_2_O_3_ [31]
SO_2_	ν_as_:1330; ν_s_: 1140	γ‐Al_2_O_3_ [31]
	Interaction Between SO_2_ and Hydroxyl Group: ∼1140	TiO_2_ [22]
	∼1140, 1150	TiO_2_ [23]
	ν_as_: 1330; ν_s_: 1149	MgO, α‐Al_2_O_3_ [24]
	ν_as_: 1326; ν_s_:1160	CeO_2_ [25]
SO_3_	1391, 1061	Pd/CeO_2_ [26]
SO_3_^2−^/HSO_3_^−^	850–1100	MgO, α‐Al_2_O_3_ [24]
	800–1150	CeO_2_ [25]
	Sulfite: 1033; Bisulfite: 1077 Monodentate Sulfite: 971, 923 Bidentate Sulfite: 1006, 806	TiO_2_ [20]
SO_4_^2−^/HSO_4_^2−^	ν_as_: 1368, 1377, 1399	TiO_2_ [30]
	1400–1300	TiO_2_ [22]
	1361, 1297, 1172, 1116, 1050, 1000	TiO_2_ [20]
	Bidentate Sulfate: 1215, 1126, 1033	TiO_2_ [27]
	1100–1200	MgO, α‐Al_2_O_3_ [24]
	S═O: 1405, 1360, 1340 S─O: 1045, 1005, 940 Bulk Sulfate: 1240, 1145, 1060, 990	CeO_2_ [25]
	Surface Species: 1400–1350 Bulk‐Like Species: 1220–1000	CeO_2_ [28]
	1420–1380 and 1290–1050	Al_2_O_3_, CuO, CuAl_2_O_4_ [29]
Sn─OH	OH_t_ Stretch: 3728–3712, 3671–3662, 3649, 3632–3625 OH_rooted_ Stretch: 3603–3589, 3555–3549, 3525–3520, 3480–3478 Deformation: 1264–1239 and 986–849	SnO_2_ [40]
Sn─O	1357–1326, 1213–1206, 1123–1119, 1062	SnO_2_ [40]
	1362, 1334, 1271, 1206, 1159, 1056	SnO_2_ [41]

Figure 2a indicates the decrease of terminal hydroxyl groups (OH_t_) (3668 and 3618 cm^−1^) and the increase of OH_rooted_ (3436 cm^−1^).^[^ ^40^ ^]^ The OH_t_ could be directly consumed by H_2_S, or the equilibrium among OH_t_, OH_rooted_, background water (still around 100 ppm in synthetic dry air), and surface oxygen species could shift to less OH_t_ formation due to the interaction between H_2_S and the surface oxygen species.^[^ ^20^, ^22^, ^23^, ^30^, ^37^, ^38^, ^39^ ^]^

The changes in the spectra region 1500–700 cm^−1^ (Figures 2b and S3d–f) provide further insights into sulfur‐containing species. Based on previous assignments (Table 1) and the principle that stronger bonds have higher vibrational frequencies, with asymmetric vibrational frequencies being even higher, we can infer the following: the asymmetric vibrations of two sulfur–oxygen double bonds (ν_as_(O═S═O)) should be present in 1400–1300 cm^−1^, its symmetric vibration (ν_s_(O═S═O)) and the vibration of one sulfur–oxygen double bond (ν(S═O)) should occur in 1210–1100 cm^−1^, and the vibration of a sulfur–oxygen single bond (ν(S─O)) in 1100–900 cm^−1^.

In the very first absorbance spectrum recorded during the exposure to 1 ppm H_2_S, i.e., the bottom curve in Figure 2b, two identified DBs at 1337 and 1289 cm^−1^ can be attributed to weakened Sn─O bonds resulting from the reaction between H_2_S molecules and surface oxygen species (adsorbed oxygen, lattice oxygen, and reactive OH_t_).^[^ ^40^, ^41^ ^]^ A broad IB at 1147 cm^−1^ corresponds to ν(S═O), and several IBs between 1000 and 900 cm^−1^ are related to ν(S─O). The simultaneous appearance of ν(S═O) and ν(S─O), without the presence of ν_as_(O═S═O) above 1300 cm^−1^, suggests the formation of SO_3_^2−^/HSO_3_^−^. Another broad IB at 1247 cm^−1^ will be discussed later. The IBs below 900 cm^−1^ have been infrequently investigated and are not in the focus of this work.

Given the high reducing ability of H_2_S, as evidenced by the ultrahigh sensor signal, a broad DB between 1400–900 cm^−1^—associated with the reduction of the Sn─O bonds—was expected as a signature of a reduced SnO_2_ surface. However, only two weak DBs (1337 and 1289 cm^−1^) are observed in the first spectrum recorded after 15 min. As H_2_S exposure continues, these DBs are even quickly compensated and replaced by several IBs (1356, 1337, and 1289 cm^−1^) (Figure 2b). The IB at 1356 is formed in the region of ν_as_(O═S═O), implying the generation of SO_2_ or SO_4_^2−^. The IB at 1289 cm^−1^ along with the sharpening IB at 1263 cm^−1^ (shifted from 1247 cm^−1^) requires further investigation. The intensifying and sharpening IB at 1147 cm^−1^, along with the broad growing IB in 1000–900 cm^−1^, suggests the ongoing generation of the SO_3_^2−^/HSO_3_^−^.

In Figure S3a,d, alongside a decrease in overall background absorbance—indicating a reduction in conduction‐band electron concentration—few differences are noted between them and those in Figure 2. This suggests the presence of persistent sticky products on the surface. A similar trend is observed in Figure S3b,c,e,f.

**Figure 3:**
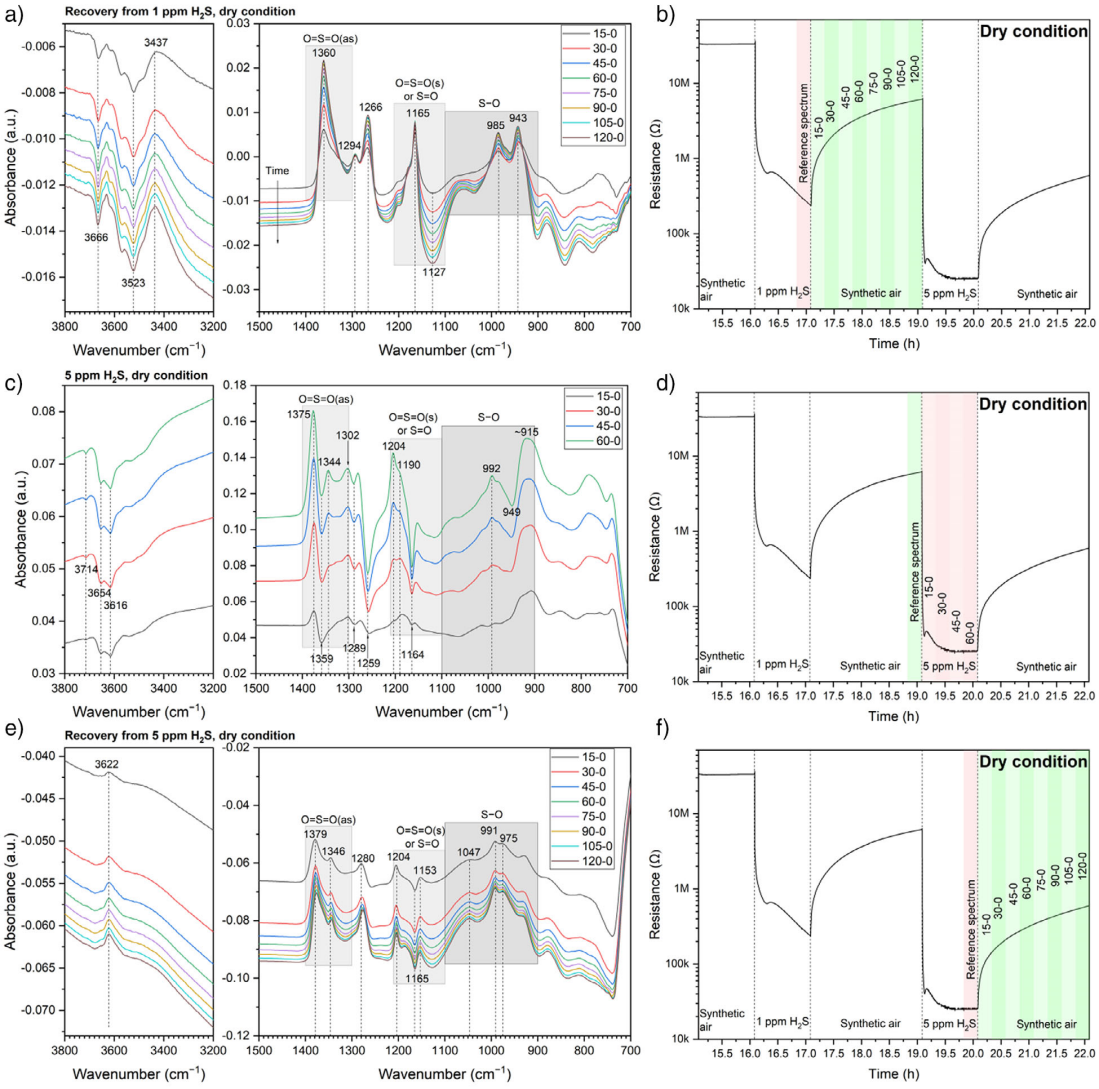
a), c), and e) Absorbance spectra under dry conditions, with b), d), and f) illustrating the acquisition timings of the reference and sample spectra.

Alternatively, by referencing the single‐channel (SC) spectra in one atmosphere to the SC spectrum before the atmosphere switch, the absorbance spectra in Figure 3 provide additional insights. During recovery from 1 ppm H_2_S (Figure 3a), OH_t_ continuously decreases, while OH_rooted_ increases with both changing slightly. The IB at 1360 cm^−1^ (SO_2_ or SO_4_^2−^) steadily grows. Weak IBs at 1165, 985, and 943 cm^−1^ suggest ongoing formation of a few SO_3_^2−^/HSO_3_^−^, despite no H_2_S being present. The only explanation is that a few intermediates are formed during H_2_S exposure, stick at the surface, and are oxidized during the re‐exposure to synthetic air. The oxidation process of the intermediates may further shift the equilibrium among hydroxyl groups from OH_t_ to OH_rooted_. Some DB below 900 cm^−1^ (Figure 3a) may result from the consumption of these intermediates. The to‐be‐explained IBs at 1294 and 1266 cm^−1^ are also observed.

During exposure to 5 ppm H_2_S, a DB around 3450 cm^−1^ is observed for the first time (Figure 3c), implying the participation of OH_rooted_ in the reaction and a significantly more reduced surface than that during 1‐ppm‐H_2_S exposure. The very low sensor resistance, along with the DB observed at 1359 cm^−1^ (the weakened Sn─O bond, also seen at 1337 cm^−1^ in Figure 2b), supports this inference. Furthermore, the previously generated SO_3_^2−^/HSO_3_^−^ is even consumed (the DBs at 1164 and 949 cm^−1^) by the highly reducing H_2_S, producing more intermediates indicated by the high plateau below 900 cm^−1^. The highly reduced surface suggests little possibility of SO_4_^2−^ generation. Thus, the IB at 1375 cm^−1^ in Figure 3c likely arises from the ν_as_(O═S═O) of SO_2_.

Figure 3e demonstrates some re‐oxidation of the surface during the recovery from 5‐ppm‐H_2_S exposure: OH_t_ and OH_rooted_ recover slightly; the intermediates are continuously oxidized (decrease below 900 cm^−1^) into SO_2_ (IB at 1379 cm^−1^).

Based on Figure 3a,c,e, it is getting clear that the IBs/DBs between 1250 and 1300 cm^−1^ correspond to the Sn─O vibrations, as indicated by their increase in air (Figure 3a,e) and clear decrease during H_2_S exposure (Figure 3c). However, this assignment indicates a counterintuitive increase of Sn─O bonds during the 1‐ppm‐H_2_S exposure by the increase at 1247–1263 cm^−1^ and the IB at 1289 cm^−1^ evolving from a DB in Figure 2b. This can occur when the H_2_S molecules are oxidized not only by the surface oxygen species but also by the O_2_ molecules in the gas phase. In other words, some O_2_ molecules in the gas phase can also take part in the oxidation of H_2_S into SO_3_^2−^/HSO_3_^−^ and SO_4_^2−^, which are adsorbed at the surface through the Sn─O bonds. This hypothesis is supported by the following DFT calculations. Besides, the DB at 1337 cm^−1^ in Figure 2b and that at 1359 cm^−1^ in Figure 3c are also assigned to Sn─O bonds. This discrepancy should be related to the surface state.

**Figure 4:**
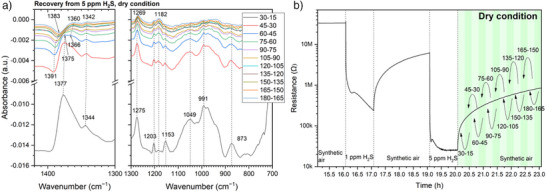
a) Absorbance spectra in dry air during the recovery from 5 ppm H_2_S with b) illustrating the acquisition timings of the reference and sample spectra.

More insight on the transformation of sulfur‐containing species can be gained from the recovery process after 5‐ppm‐H_2_S exposure by the spectra in Figure 4. The bottom curve (30–15) shows a notable but asymmetric IB at 1377 cm^−1^ accompanied by a left‐side emerging DB and a right‐side shoulder. The DB becomes visible at 1391 cm^−1^ after 15 min, develops over time, and shifts to 1383 cm^−1^. The shoulder becomes evident at 1366 cm^−1^ after 30 min and shifts to 1360 cm^−1^. As the surface is being oxidized in air in this period, indicated by the increase of Sn─O bonds (1275–1269 cm^−1^), the generated SO_2_ during 5‐ppm‐H_2_S exposure can transform into SO_3_^2−^/HSO_3_^−^ and then into SO_4_^2−^, demonstrated by IBs at 1203, 1182, and 1153 cm^−1^ (ν(S═O)) and the broad IBs at 1049 and 991 cm^−1^ (ν(S─O)). Thus, the DB (1391–1383 cm^−1^) should be due to further transformation of SO_2_. It is not observed at the beginning, likely because, at that time, SO_2_ is meanwhile supplemented by the oxidation of intermediates. As the supply from the intermediates slows, the IB (originally at 1377 cm^−1^) degrades and then disappears. The IB at 1366–1360 cm^−1^ is attributed to the SO_4_^2−^, and the one at 1344 cm^−1^ to Sn─O.

The absorbance spectra recorded during the recovery from 1 ppm H_2_S under dry conditions (Figure S4) are analyzed similarly and show different results from Figure 4. No DB around 1391 cm^−1^ but only an IB at 1361 cm^−1^ is observed because SO_2_ is not the main product formed during 1‐ppm‐H_2_S exposure. The continuous generation of SO_4_^2−^ during exposure to and recovery from H_2_S accounts for the IBs at 1356–1362 cm^−1^ in Figures 2, 3, and S4a.

#### DRIFTS Under Humid Conditions

Further DRIFTS results obtained under humid conditions support the hypotheses presented above and offer insights into how humidity affects the surface chemistry of sulfur‐containing species.

**Figure 5:**
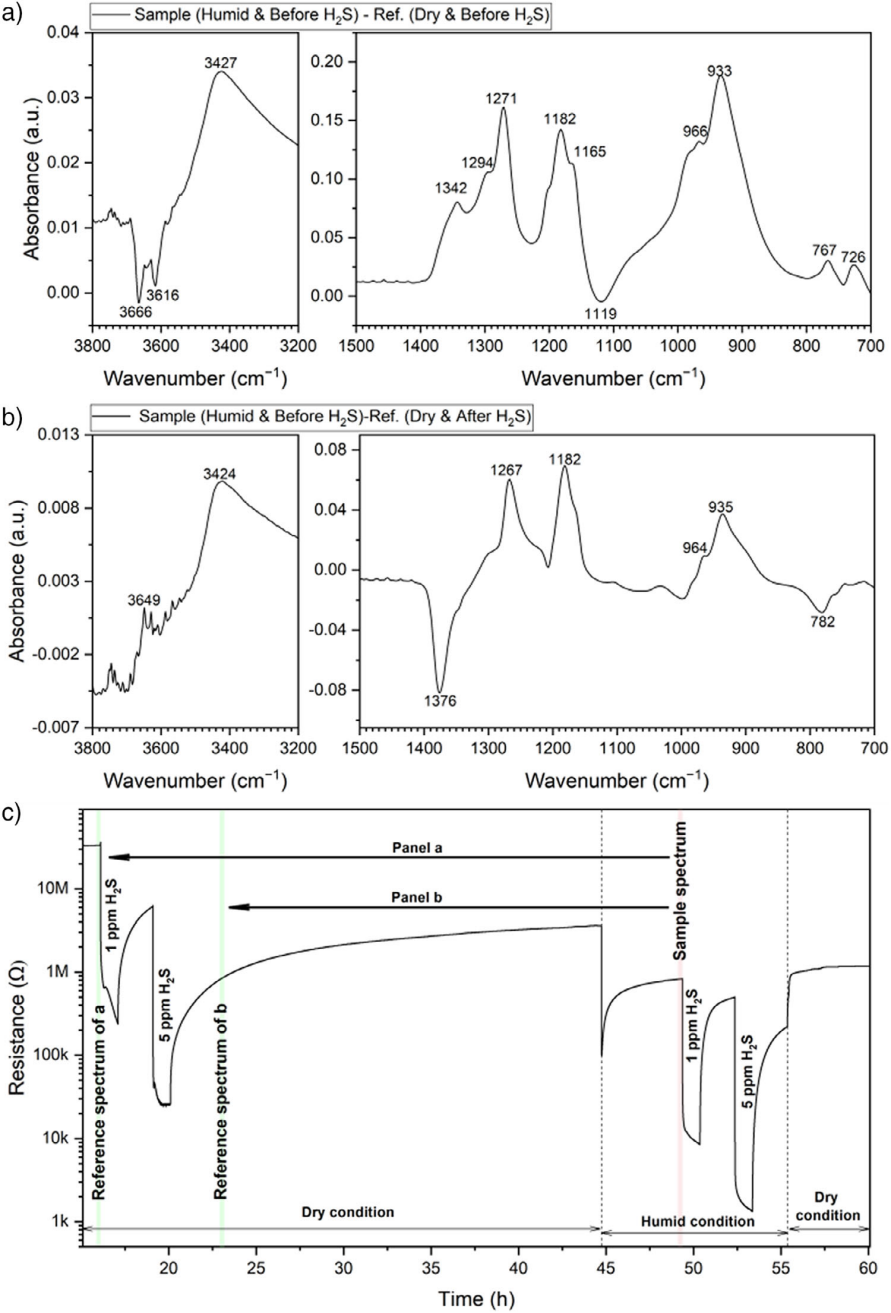
a) and b) Absorbance spectra in humid air before H_2_S injection with c) illustrating the acquisition timings of the reference and sample spectra.

Figure 5 shows two different absorbance spectra recorded in humid conditions before the H_2_S exposure. Figure 5a identifies the surface species remaining at the surface, while Figure 5b again reveals the further transformation of the H_2_S reaction products. In Figure 5a, the IBs corresponding to vibrations of S═O (1342, 1182, and 1165 cm^−1^) and ν(S─O) (966 and 933 cm^−1^) suggest a significant presence of SO_3_^2−^/HSO_3_^−^ and SO_4_^2−^ species. In Figure 5b, a deep DB at 1376 cm^−1^ likely results from the further transformation of SO_2_, as described in relation to the results in Figure 4. The OH_rooted_ has been added to the surface (broad IB around 3427 cm^−1^ in Figure 5a) mainly by the previous H_2_S exposure and some by the humidity (IBs above 3000 cm^−1^ in Figure 5b). The OH_t_ has not fully recovered (two sharp DBs at 3666 and 3616 cm^−1^ in Figure 5a).

**Figure 6:**
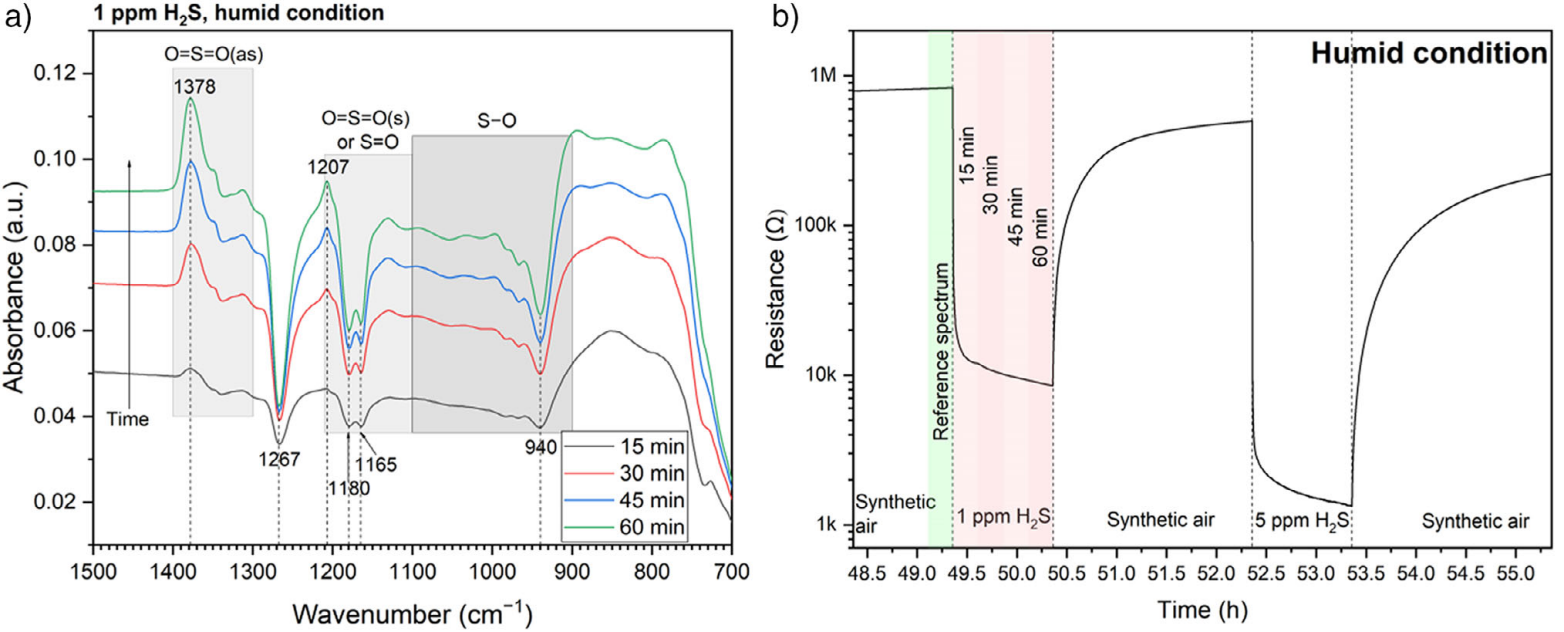
a) Absorbance spectra during 1‐ppm‐H_2_S exposure under humid conditions with b) illustrating the acquisition timings of the reference and sample spectra.

**Figure 7:**
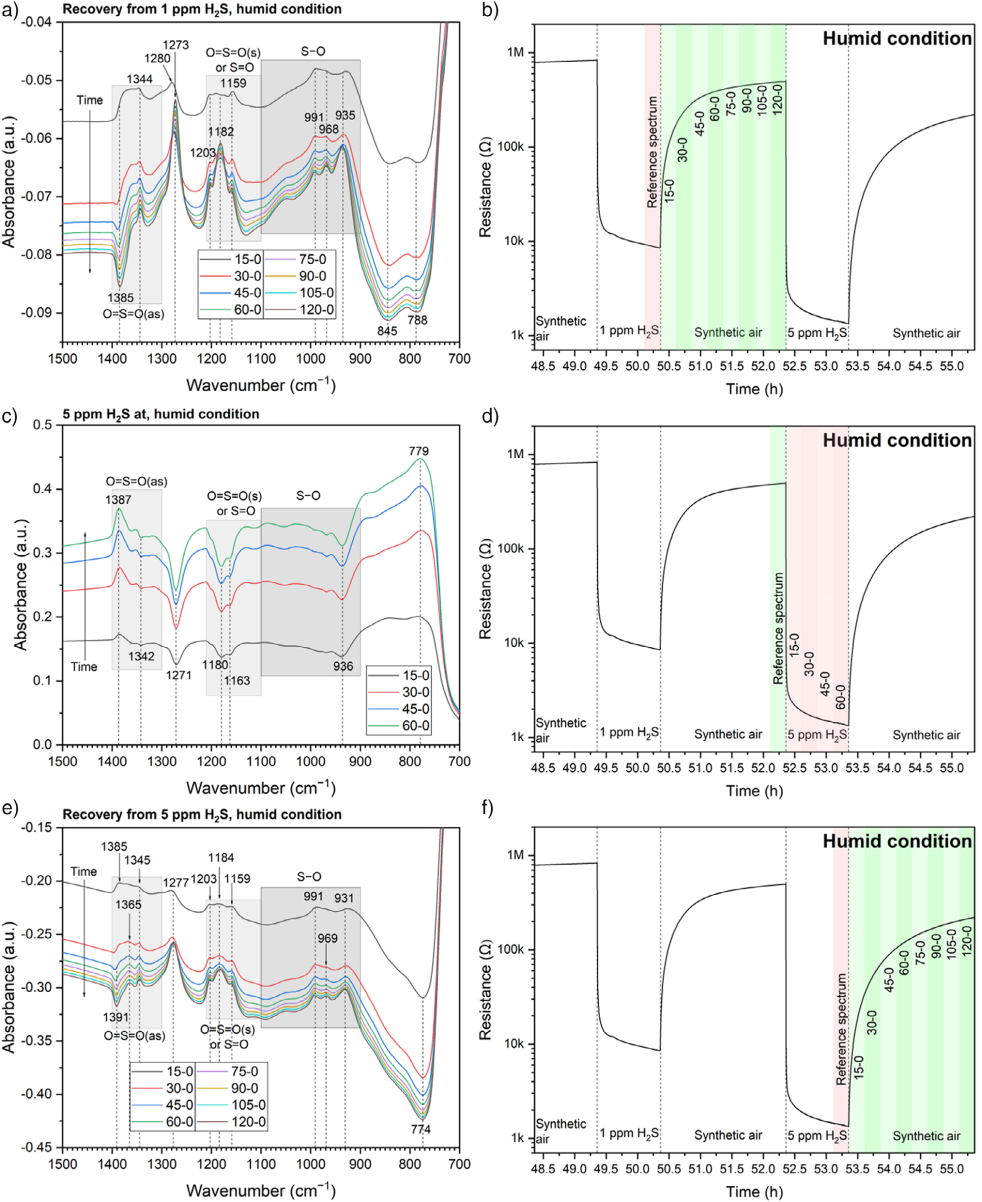
a), c), and e) Absorbance spectra under humid conditions with b), d), and f) the acquisition timings of the reference and sample spectra.

DRIFTS results in humid air are shown in Figures 6, 7, and S5, S6. The IB at 1378 cm^−1^ in Figure 6a is attributed to SO_2_, for the same reason given to Figure 3c. The surface remains significantly reduced as indicated by the weakening Sn─O bond (DB at 1267 cm^−1^), the consumption of SO_3_^2−^/HSO_3_^−^ (DBs at 1180, 1165, and 940 cm^−1^), and the formation of intermediates (high plateau below 900 cm^−1^). Figure 7a indicates that the surface is re‐oxidized during recovery from 1 ppm H_2_S, as evidenced by the increase of IBs corresponding to Sn─O bonds (1273–1280 cm^−1^) and the regeneration of SO_3_^2−^/HSO_3_^−^ (1159, 968, and 935 cm^−1^). The DB at 1385 cm^−1^ results from the further transformation of SO_2_, and intermediates are consumed (indicated by the decrease below 900 cm^−1^). Subsequent exposure to and recovery from 5 ppm H_2_S in Figures 7c–f, S5, and S6 suggest that the surface reactions are somewhat reversible. However, SO_4_^2−^ appears to accumulate, as the broad IB between 1340 and 1380 cm^−1^ persists during recovery without a corresponding DB during H_2_S exposure.

By now, the assignment of infrared bands for sulfur‐containing species on SnO_2_ is also clear: the ν_as_(O═S═O) of adsorbed SO_2_ surface locates in 1400–1370 cm^−1^, while that of SO_4_^2−^ in 1370–1340 cm^−1^ (some overlap with Sn─O). The ν_s_(O═S═O) and ν(S═O) should be in 1210–1100 cm^−1^, and ν(S─O) in 1100–900 cm^−1^.

### DFT Calculations

#### Reaction Path

**Figure 8:**
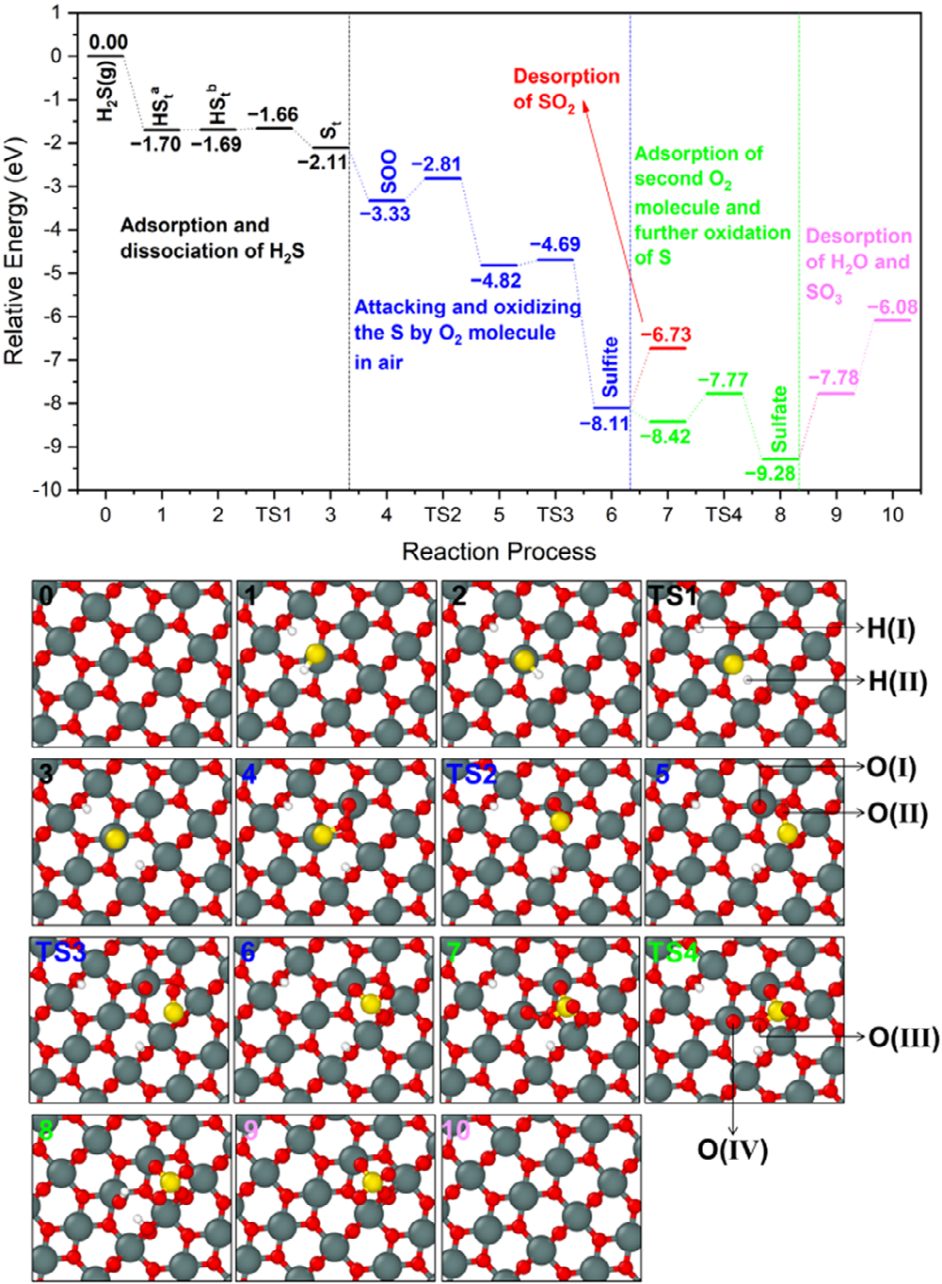
Reaction route and energy profile of H_2_S at stoichiometric SnO_2_ (110) surface (The panels below show the atom structures. TS means transition state. The white is H, red O, yellow S, and grey Sn. The atoms labeled with Roman numbers correspond to the atoms for Bader charge analysis in Table 2).

This section aims to reveal the reaction pathway from H_2_S to surface SO_3_^2−^ and SO_4_^2−^. By a simple relaxation, an H_2_S molecule positioned 3 Å above the SnO_2_ (110) surface is adsorbed and spontaneously dissociates, releasing 1.70 eV of energy. The SnO_2_ (110) crystal plane has been demonstrated to take most part of the exposed surface of SnO_2_ nanoparticles and has been extensively investigated by DFT simulation.^[^ ^37^, ^42^, ^43^, ^44^ ^]^ The out‐of‐plane oxygen (or the bridge oxygen) at the SnO_2_ (110) extracts one H atom from the H_2_S molecule, generating OH_rooted_, while the sulfur bonds to the surface Sn, forming a terminal sulfhydryl group (SH_t_) (Figure 8, step 1). The generated SH_t_, influenced by the nearest out‐of‐plane oxygen, loses the other H atom, becoming a terminal sulfur atom (S_t_) and generating another OH_rooted_ (Figure 8, step 3). The OH_rooted_ contributes to the IBs observed around 3450 cm^−1^ in DRIFTS. Unlike the double‐coordinated sulfur on the wrinkled amorphous SnO_2_ surface,^[^ ^8^ ^]^ herein, the S_t_ is single‐coordinated on the well‐crystallized SnO_2_ (110) surface.

The single‐coordinated S_t_ is highly reactive and easily attacked by an O_2_ molecule, which is present at much higher concentrations than H_2_S in the measurement atmosphere, i.e., synthetic air. This reaction generates SOO species and releases 1.22 eV of energy. Besides, the O_2_ molecule can also attack the SH_t_, making it rotate, dissociate, and produce SOO species (Movie S1). The spontaneous dissociation of H_2_S and the following attack by O_2_ always release considerable energy by crossing a negligible energy barrier. This demonstrates the high reactivity of H_2_S on the SnO_2_ surface and explains why H_2_S/HS is not observed in the DRIFTS.

It seems reasonable that the so‐generated SOO transforms into SO_2_ by breaking the O─O bond. However, the SO_2_ molecule is unstable at the surface, and it spontaneously interacts with out‐of‐plane oxygen to form the SO_3_^2−^ (Movie S2), complicating the identification of a transition state (TS) between SOO and SO_2_ through DFT calculations. Other potential intermediate steps from SOO to SO_3_^2−^ are simulated (Figure 8: steps 4–6). The initial rotation of SOO and its interaction with the out‐of‐plane oxygen are illustrated, with step 6 being a highly exothermic reaction that releases 3.19 eV. In the resulting SO_3_^2−^, one oxygen atom comes from the surface (out‐of‐plane oxygen), while the other two are from the O_2_ molecule. The capture of an O_2_ molecule from the gas phase to form SO_3_^2−^ explains the Sn─O IB (1289 and 1263 cm^−1^) recorded during the exposure to 1 ppm H_2_S (Figure 2b). The very short lifetime of SO_2_ explains why only SO_3_^2−^/HSO_3_^−^ are detected by DRIFTS at the beginning of H_2_S injection.

Breaking down SO_3_^2−^ to form SO_2_ requires 1.38 eV. Alternatively, it can be further oxidized by another of the numerous O_2_ molecules in the air attacking the surface bridging one surface Sn and the S atoms (Figure 8, step 7), further explaining the recorded Sn─O peak (1289 and 1263 cm^−1^) in Figure 2b. After the dissociation of the second O_2_ molecule, the sulfite transforms into a sulfate, and the O bonded to the surface Sn converts into OH_t_ by taking the H atom from OH_rooted_ (Figure 8, step 8). This process requires an activation energy of 0.65 eV and releases 0.86 eV. The relatively high activation energy explains the DRIFTS observation of co‐presence of SO_3_^2−^/HSO_3_^−^ and SO_4_^2−^ at the surface. SO_4_^2−^ seems unlikely to decompose to SO_3_ because of the energy requirement (1.70 eV). OH_t_ and OH_rooted_ can desorb as water molecules, also requiring high energy of 1.50 eV. Under practical experimental conditions, the water desorption‐adsorption equilibrium is influenced by the operational temperature and ambient humidity.

The detailed reaction processes are illustrated as Equations 5:

(5a) $$H_{2}S(g)+\mathrm{Sn}O_{2}+O_{2}\left(g\right)\to \mathrm{Sn}{\left({\mathrm{OH}}_{\mathrm{rooted}}\right)}_{2}+\mathrm{SO}O_{\mathrm{ads}}$$

(5b) $$2\mathrm{SO}O_{\mathrm{ads}}+\mathrm{Sn}O_{2}\to \mathrm{Sn}{\left({\mathrm{SO}}_{3}\right)}_{2}$$

(5c) $$\mathrm{Sn}{\left({\mathrm{SO}}_{3}\right)}_{2}+O_{2}\left(g\right)\to \mathrm{Sn}{\left({\mathrm{SO}}_{4}\right)}_{2}$$

(5d) $$2\mathrm{Sn}{\left({\mathrm{OH}}_{\mathrm{rooted}}\right)}_{2}+O_{2}\left(g\right)\to 2\mathrm{Sn}O_{2}+2H_{2}O\left(g\right)$$

They can be summarized, if all the H_2_S is eventually oxidized into sulfate adhered to and water molecule desorbed from the surface, as Equation 6:

(6) $$2H_{2}S(g)\;+\;\mathrm{Sn}O_{2}+4O_{2}\left(g\right)\to \mathrm{Sn}{\left({\mathrm{SO}}_{4}\right)}_{2}+2H_{2}O(g)$$

The oxidation of H_2_S on the SnO_2_ (110) surface releases 8.11 eV when producing SO_3_^2^⁻ and 9.28 eV for SO_4_^2^⁻. From the energy perspective, the SO_3_^2^⁻ and SO_4_^2^⁻ are more likely to accumulate on the SnO_2_ surface with continuous H_2_S exposure. This accumulation aligns with the findings obtained by the DRIFTS measurements and confirms the ex situ XPS characterization by Lee et al.^[^ ^9^ ^]^

Equations 5 describe the adsorption and reaction of H_2_S on the stoichiometric SnO_2_ (110) surface, assuming a continuous supply of O_2_ molecules and neglecting the roles of oxygen vacancies and water molecules (or hydroxyl groups). This scenario represents an initial highly oxidized SnO_2_ surface in dry conditions, capable of oxidizing low concentrations of H_2_S into sulfite and sulfate with minimal SO_2_ formation, aided by abundant gas‐phase O_2_ molecules.

For a highly reduced SnO_2_ surface and with the presence of the generated sulfite and sulfate, the reaction routes and products can become more complex. DFT calculations reveal that one sulfate nearby can stabilize the SO_2_ molecule at the surface (Figure S7). This research aims to clarify the production of sulfite and sulfate and their effects on the conductivity. Instead of simulating other complex reactions, the focus shifts to the charging effects of different adsorbates.

#### Charging Effects

To relate the resistance variation of the SnO_2_ device to the above simulated reactions, the charging effect of each reaction step is analyzed. The SnO_2_ sample used here is an n‐type semiconductor with electrons being the dominant free charge carriers. Thus, electron donation enhances the conductivity (resistance decreases), whereas electron trapping reduces the conductivity (resistance increases). Table 2 lists the Bader charge of adsorbates at steps of reaction route in Figure 8.^[^ ^45^ ^]^

**Table 2:** Bader charge analysis of adsorbates.

Step	Bader Charge/(*e*)							
	Total	H(I)	H(II)	S	O(I)	O(II)	O(III)	O(IV)
1	**0.638**	1.000	−1.167	0.805	Not Adsorbed Yet			
2	**0.742**	0.999	−1.242	0.985				
3	**1.368**	1.000	1.000	−0.632				
4	**1.294**	0.999	1.000	0.648	−0.451	−0.902	Not Adsorbed Yet	
5	**1.917**	0.745	0.754	2.400	−0.654	−1.328		
6	**1.554**	0.999	0.602	3.544	−1.800	−1.790		
7	**2.485**	0.999	1.000	5.880	−1.842	−1.884	−1.154	−0.514
8	**0.659**	1.000	1.000	5.879	−1.795	−1.912	−1.925	−1.587
9	**0.265**	Desorbed		5.876	−1.765	−1.919	−1.927	Desorbed

Note: (1) The indicated values are differences between the ideal number of valence electrons and the integral charge calculated by the Bader program. (2) The atoms here are labeled in Figure 8. (3) For the charge effect of the sulfite, only the one S atom and the two O atoms, i.e., O(I) and O(II), of the captured O_2_ are analyzed. At step 6, the sum (3.544 − 1.800 − 1.790) is −0.046. Same for the sulfate; its Bader charge is 0.247 at step 8 and 0.265 at step 9, the sum of the S, the O(I), O(II), and O(III).

The calculations indicate that the majority of the electron donation effect originates from OH_rooted_ (Table 2, steps 3–7), which are primarily responsible for the significant decrease in resistance. Even at step 8, when one OH_rooted_ transforms into OH_t_, the total Bader charge, calculated as the sum of H(I), H(II), and O(IV) (1.000 + 1.000 − 1.587), remains at 0.413. The OH_rooted_ is consistently observed as a broad IB band around 3450 cm⁻¹ in DRIFTS, even after 2 h of dry air flushing (Figures 2 and S3), because 1.50 eV is required for the desorption of the OH_rooted_ and OH_t_ as water molecules. The OH_rooted_ leads to a slow recovery process.

The sulfate (0.265) contributes to the poor recovery (Table 2, step 9), while the sulfite (−0.046) does not (Table 2, step 6). Since both sulfite and sulfate strongly adhere to the surface, and the sulfite is eventually oxidized into sulfate, the accumulation of the electron‐donating sulfate causes the baseline resistance to decrease, preventing it from returning to its initial value.

## Discussions

The whole picture of H_2_S sensing mechanism and sulfur poisoning effect is clear based on the DRIFTS and DFT results. The original SnO_2_ surface in an air atmosphere at 300 °C is of highly oxidizing ability, which can oxidize the initially injected 1 ppm H_2_S into SO_3_^2−^/HSO_3_^−^ and SO_4_^2−^ with little stabilized SO_2_. Some OH_t_ are consumed during the process and never compensated. DFT calculations verify the high tendency of H_2_S into sulfite and sulfate at the oxidizing surface.

After the initial H_2_S exposure, the surface is reduced and difficult to recover because of the sticky products, such as OH_rooted_, sulfite, sulfate, and a few intermediates. The reduced surface as well as the higher concentration in the subsequent exposure make the products of 5 ppm H_2_S different. The SO_2_ seems to be stabilized at the surface, and many more intermediates are generated. The SO_2_ molecules and the intermediates keep evolving during the long‐period recovery, and the sulfite and sulfate accumulate.

With the large amount of sticky products at the surface, the further measurement under humidity conditions is more reversible, but with further accumulation of sulfate.

The charge effect analysis demonstrates the ultrahigh responses to H_2_S with remarkable resistance decreases are mainly attributed to the generated OH_rooted_ which is also some responsible for the slow recovery process. The sticky sulfite and sulfate are the root cause of the poisoning effect, especially the sulfate, which is the ultimate product and possesses an electron‐donating effect, making the baseline resistance continuously shift downwards.

It is worth noting that the commercially utilized SnO_2_ gas sensors aiming to other target gases can also be poisoned if they are exposed to H_2_S, SO_2_ or other sulfur‐containing volatile organic compounds (VOCs), and the same for other SMOX‐based gas sensors.

Increasing the temperature can make the Sn(SO_4_)_2_ decompose, but not until 500 °C,^[^ ^46^ ^]^ at which the gas sensor itself can be ignitor. Instead, WO(SO_4_)_2_ begins to decompose around 300 °C. Also as one of the most investigated SMOX sensing material, WO_3_ exhibits distinct surface chemistry from SnO_2_, according to our previous research in the water adsorption and reaction.^[^ ^37^, ^38^, ^40^ ^]^ WO_3_ can be a promising candidate for H_2_S sensing.

## Conclusions

The DRIFTS and DFT results provide compelling evidence of the sulfur poisoning effect of H_2_S on SnO_2_ gas sensor. The root cause of the poisoning effect is the formation of sticky sulfite and sulfate species. The ultimate product, sulfate, has an electron‐donating effect, leading to a prolonged lack of recovery. The detailed reactions of H_2_S on the initial high‐oxidizing surface as well as on the partially reduced and contaminated surface are also illustrated. The reaction processes, energy profile, and the charge effect of each product are thoroughly analyzed by DFT. The findings regrettably indicate that the SnO_2_ is not a suitable sensing material for H_2_S. To overcome this issue requires alternative SMOX materials with different surface chemistries. The methods and findings of this research are also helpful for investigating and understanding other metal‐sulfur–oxygen systems, such as desulfurization catalysts, denitration catalysts, and solid oxide fuel cells.

## Supporting Information

Experimental and computational details as well as some complementary materials are available free of charge via the internet at http://. The authors have cited additional references within the Supporting Information.^[^ ^36^, ^37^, ^42^, ^43^, ^44^, ^45^, ^47^, ^48^, ^49^ ^]^

## Conflict of Interests

The authors declare no conflict of interest.

## Supporting information



Supporting Information

Supporting Information

Supporting Information

## Data Availability

The data that support the findings of this study are available on request from the corresponding author. The data are not publicly available due to privacy or ethical restrictions.
