# Supplementary material for: H2S Sensing with SnO2‐Based Gas Sensors: Sulfur Poisoning Mechanism Revealed by Operando DRIFTS and DFT Calculations
Source: Angew Chem Int Ed Engl. 2025 Apr 4;64(23):e202504696. doi: 10.1002/anie.202504696 (PMC12124429; doi:10.1002/anie.202504696)
Supplement: Supplementary file 1 — Supporting Information [file ANIE-64-e202504696-s001.docx]

***Supporting Information***

**H_2_S Sensing with SnO_2_-Based Gas Sensors: Sulfur Poisoning Mechanism Revealed by Operando DRIFTS and DFT Calculations**

Tingqiang Yang,^[a,b,c,1]^ Matthias Boepple,^[a,b,1]^ Anne Hémeryck,*^[c]^ Antoine Jay,^[c]^ Sara Karwounopoulos,^[a,b]^ Udo Weimar,^[a,b]^ Nicolae Barsan*^[a,b,d]^

^[a]^ Institute of Physical and Theoretical Chemistry (IPC), University of Tuebingen, Auf der Morgenstelle 15, D-72076, Tuebingen, Germany

^[b]^ Center for Light-Matter Interaction, Sensors & Analytics (LISA^+^), University of Tuebingen, Auf der Morgenstelle 15, D-72076, Tuebingen, Germany

^[c]^ LAAS-CNRS, Université de Toulouse, CNRS, F-31555, Toulouse, France

^[d]^ International Research Organization for Advanced Science and Technology (IROAST), Kumamoto University, Kumamoto, Japan

^[1]^ These authors contribute equally to this work.

* Corresponding authors: nb@ipc.uni-tuebingen.de; anne.hemeryck@laas.fr

**Experimental Details**

The sensor device was fabricated by screen printing using commercially available SnO_2_-powder (99.99 %, trace metals basis, Sigma Aldrich), labelled SA. A home-made chamber with KBr window allows simultaneous recording of the DRIFTS signal by a Fourier-transform Infrared spectrometer (Bruker Vertex 70V) during the resistance measurement by a digital multimeter (Keithley 199 or Keithley 2000). The device could be heated to a given temperature by an external power supply (Agilent E3630A); here it was set to 300 °C. The carrier gas was synthetic air (~21 % O_2_, ~79 % N_2_).


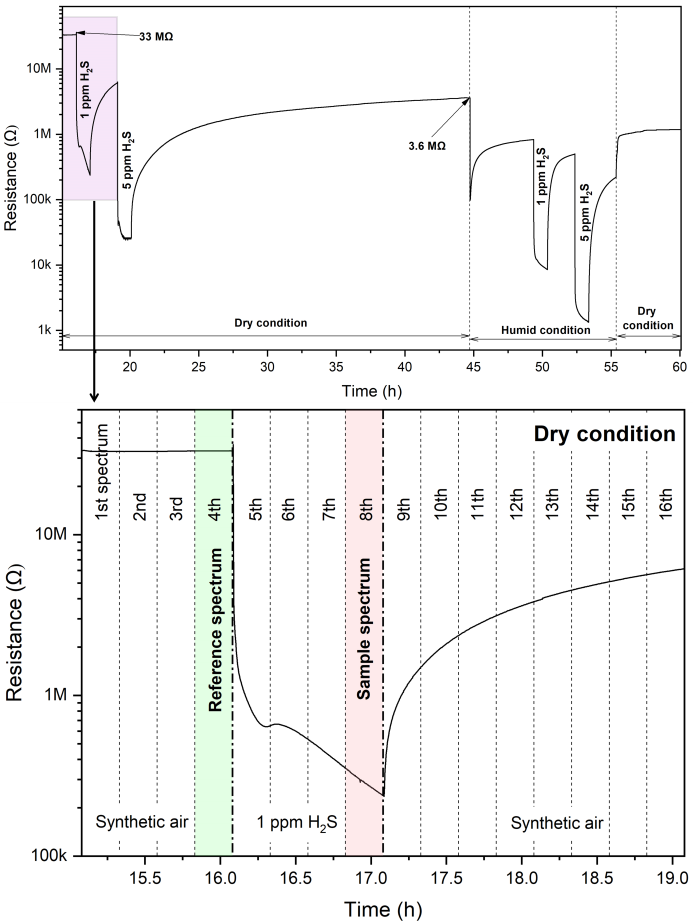


**(a)**

**(b)**

Figure S1 (a) Overall resistance curve of SnO_2_ sensor device during H_2_S DRIFTS measurement in dry and humid air with H_2_S concentration of 1 and 5 ppm at 300 °C, (b) zoom of the beginning of penal (a) showing the acquisition of a single-channel spectrum every 15 min and the pick-up of the reference and sample spectra.

The DRIFTS measurement was performed under both dry (0 r.h.% @ 25 °C) and humid (10 r.h.% @ 25 °C) conditions with H_2_S concentrations of 1 and 5 ppm, as shown in Figure S1a. Each single-channel (SC) infrared spectrum was measured by performing 1024 scans over 15 minutes with a resolution of 1 cm^−1^. This allowed us to obtain, for example, during the first 4 hours (Figure S1b): 4 SC spectra under initial air conditions, 4 at 1 ppm H_2_S, and 8 during the recovery phase from 1 ppm H_2_S. The fourth (or the last) spectrum in the initial dry air exposure was taken as the reference, and the absorbance amplitude for subsequent sample spectra was calculated relative to this reference using Equation S1. Different reference spectrum can be selected..

$\text{A= }-\text{log (}\frac{\text{SC}_{\text{Sample}}}{\text{SC}_{\text{Reference}}}\text{)}$ Equation S1

**Computational Details**

The periodic DFT calculations were performed by the plane-wave program Quantum Espresso 6.6 using the general gradient approximation (GGA) in the form of a Perdew-Burke-Ernzerhof (PBE) functional.^[^[^1^](#_ENREF_1)^]^ The projector-augmented wave (PAW) potentials from the website were used to represent the core electrons of each type atom in the system.^[^[^2^](#_ENREF_2)^]^ The size of the plane wave basis set was limited with a cutoff energy of 75 Ry. The Brillouin-zone integrations were performed using a 6 × 6 × 6 Monkhorst-Pack grid for relaxation of both lattice parameters and atom positions of SnO_2_ primitive cell. A supercell of SnO_2_ (110) surface was built based on the relaxed primitive cell. The lattice parameters of the supercell are a = b = 18.866 Å, c= 27.992 Å and α = β = 90°, γ = 92.953°. It possesses 384 atoms (256 O and 128 Sn) placed in 8 O layers and 4 Sn-O layers. The atoms in the bottom O layer and Sn-O layer are fixed at their bulk position, while others can be relaxed to simulate surface. A 15 Å vacuum layer is added in the ***c*** direction to avoid boundary effect induced by the periodic lattice, as shown in Figure S2. For the calculation of the surface supercell, the Brillouin zone has been sampled at zone center only (Gamma point). Energy barriers have been calculated using the Activation Relaxation Technique coupled with DFT using a 0.05 eV/Å threshold of force.^[^[^3^](#_ENREF_3)^]^

Charge transfer was calculated by Bader code.^[^[^4^](#_ENREF_5)^]^ Herein, the indicated values in Table 2 are differences between the ideal number of valence electrons and the integral charge calculated by the Bader program. The Bader charge of H(Ⅰ) (Table 2, step 1) is 1.000, which is obtained by subtracting its valence electron number (1.000) with the Bader program integrated charge (0.000). Positive values suggest electron donation.

**(a)**

**(b)**


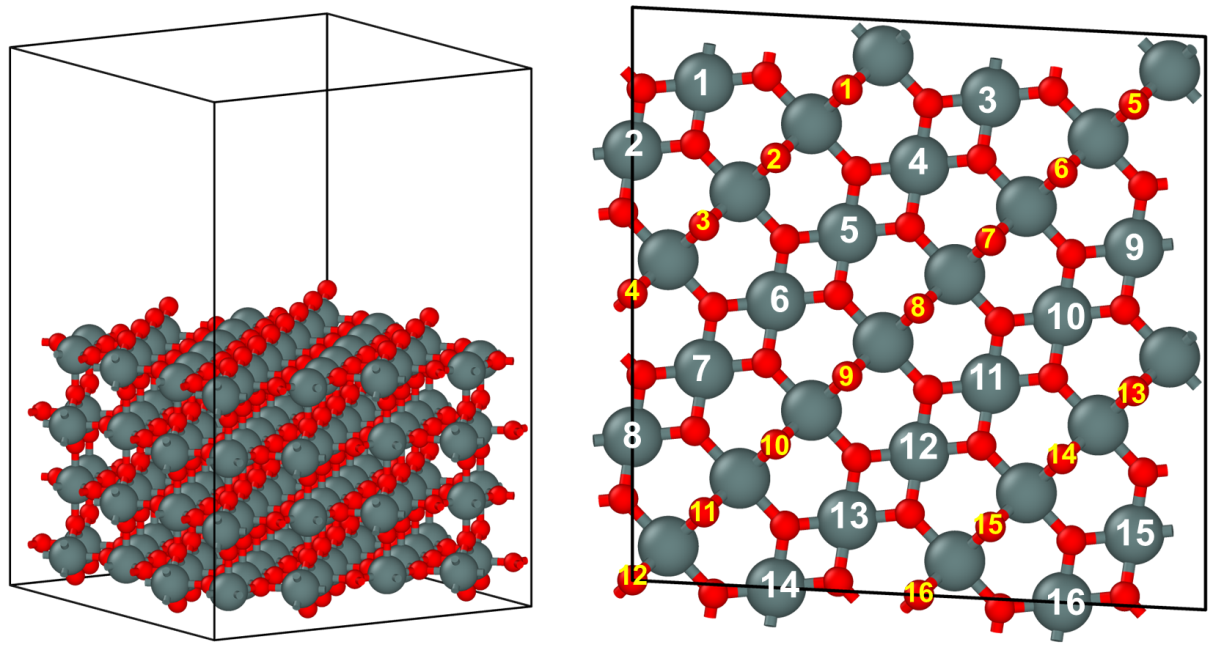


Figure S2 SnO_2_ (110) surface: (a) Supercell for the simulation and (b) the top layer with 16 out-of-plane oxygen in yellow number and 16 exposed tin atoms in white number.

The focus is on SnO_2_ (110) crystal plane, which has been demonstrated to take most part of the exposed surface of SnO_2_ nanoparticles and has been extensively investigated by DFT simulation.^[^[^5^](#_ENREF_6)^]^ The adsorption energy (E_ads_), activation energy (*i.e.* energy barrier, E_a_), and reaction energy (E_react_) are defined by Equations S2.

E_ads_ = E_SnO2 (110) + molecule_ – E_SnO2 (110)_ – E_molecule_ Equation S2a

E_a_ = E_TS_ – E_reactant_ Equation S2b

E_react_ = E_product_ – E_reactant_ Equation S2c

Herein, the E_SnO2 (110) + molecule_ represents the total energy of SnO_2_ (110) surface with an adsorbed molecule, and E_SnO2 (110)_, E_molecule_ respectively means the energy of the surface and the molecule. E_SP_ is the energy of the transition state (TS), and E_product_, E_reactant_ are energies of products and reactants respectively.

Table S1 Resistance and sensor signal in dry and humid air.

| Condition | Stages | Resistance /Ω | Sensor signal |
| --- | --- | --- | --- |
| Dry | Original | 33,300,000 | 1 |
|  | 1 ppm H_2_S | 236,000 | 141 |
|  | 5 ppm H_2_S | 26,000 | 1281 |
| Humid | Original | 831,460 | 1 |
|  | 1 ppm H_2_S | 8500 | 98 |
|  | 5 ppm H_2_S | 1300 | 640 |

Note: (1) The ‘Original’ resistance in dry and humid air condition is the stable resistance before H_2_S injection, and the resistance in 1 ppm and 5 ppm H_2_S under both dry and humid conditions is the last resistance before re-exposure to air. (2) The ‘Sensor signal’ is calculated by dividing the ‘Original’ resistance with the resistance in 1 and 5 ppm H_2_S.

**(a)**

**(d)**


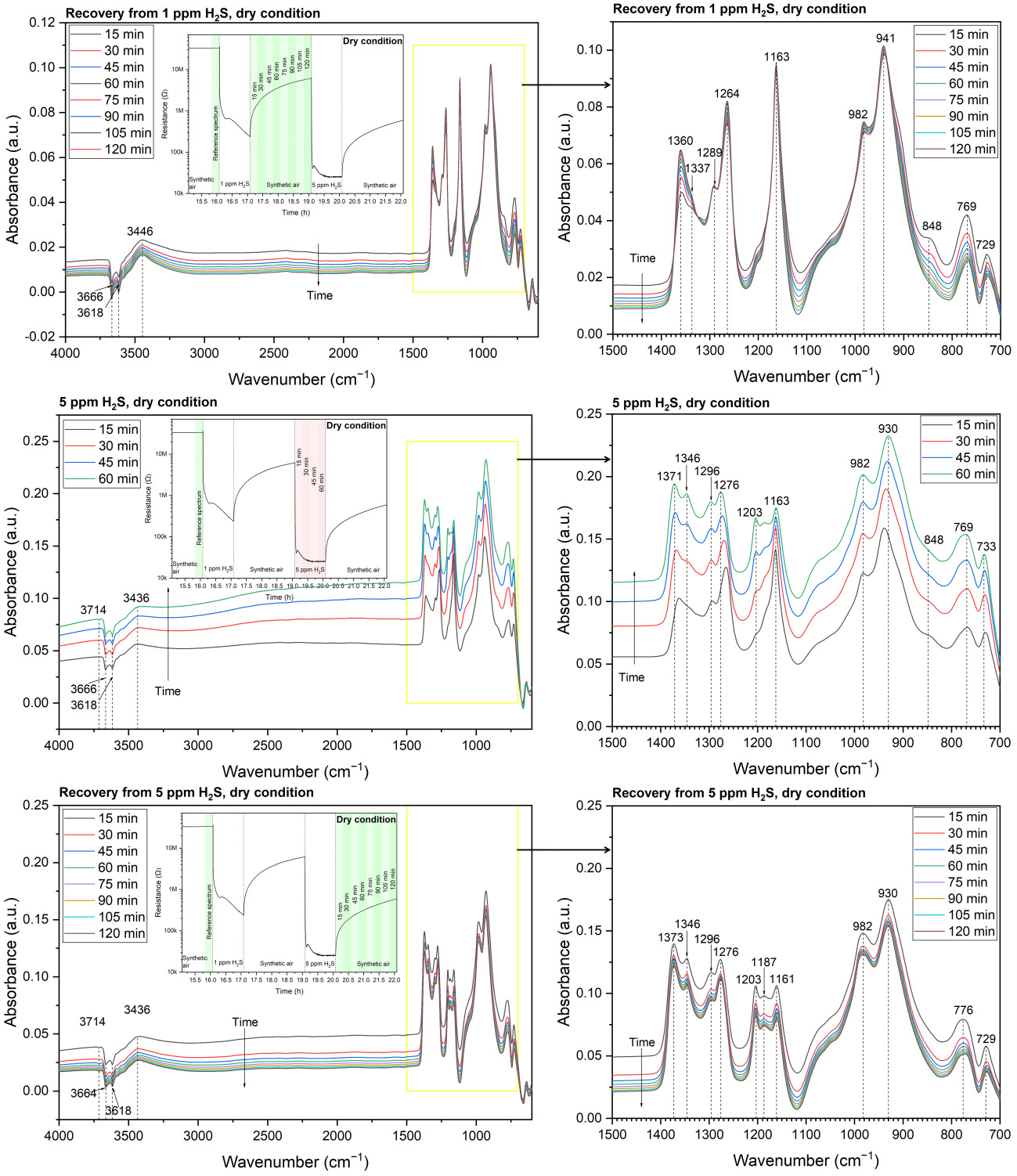


**(f)**

**(c)**

**(e)**

**(b)**

Figure S3 Absorbance spectra during the (a, d) recovery from 1 ppm H_2_S, as well as (b, e) exposure to and (c, f) recovery from 5 ppm H_2_S at 300 °C in synthetic dry air. The panels (a-c) show the whole measurement range 4000-600 cm^−1^ and the panels (d-f) 1500-700 cm^−1^. The insets in panel (a-c) show the acquisition timings of the reference and sample spectra.


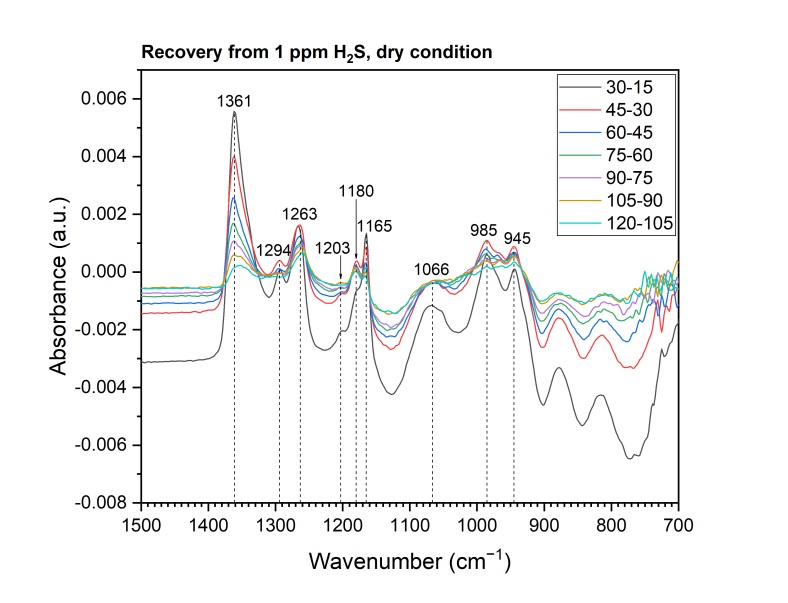

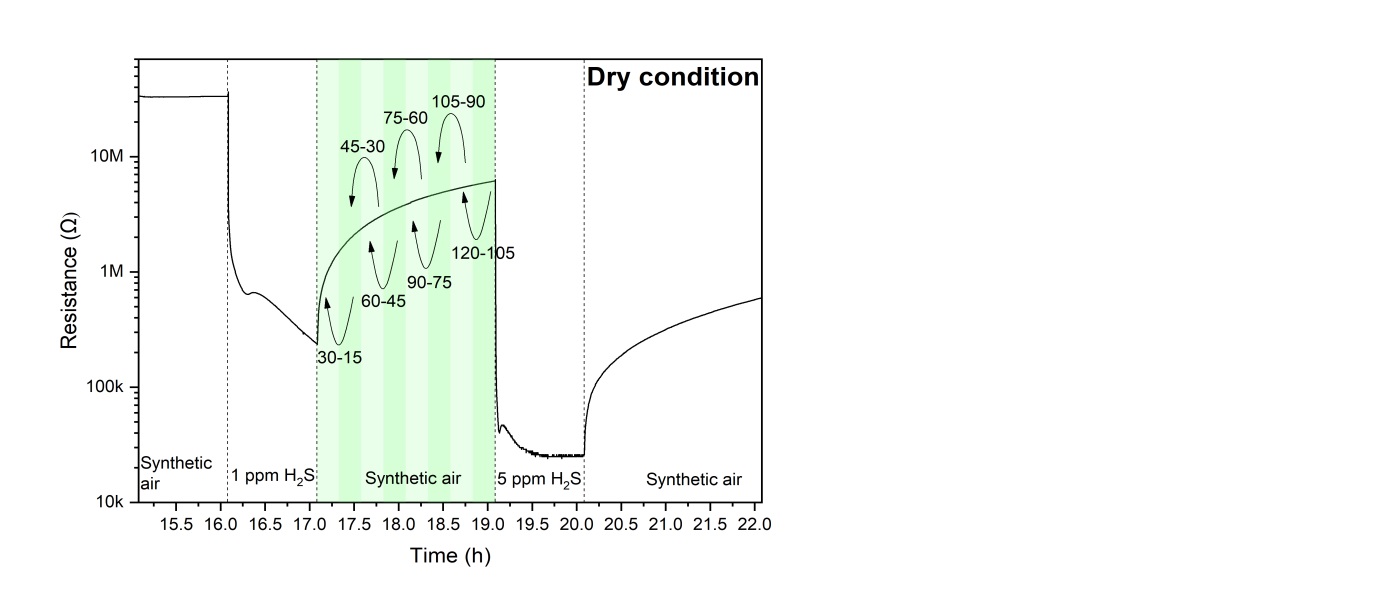


**(a)**

**(b)**

Figure S4 (a) Absorbance spectra in dry condition during the recovery from 1 ppm H_2_S, (b) the pick-up of the sample and reference spectra.


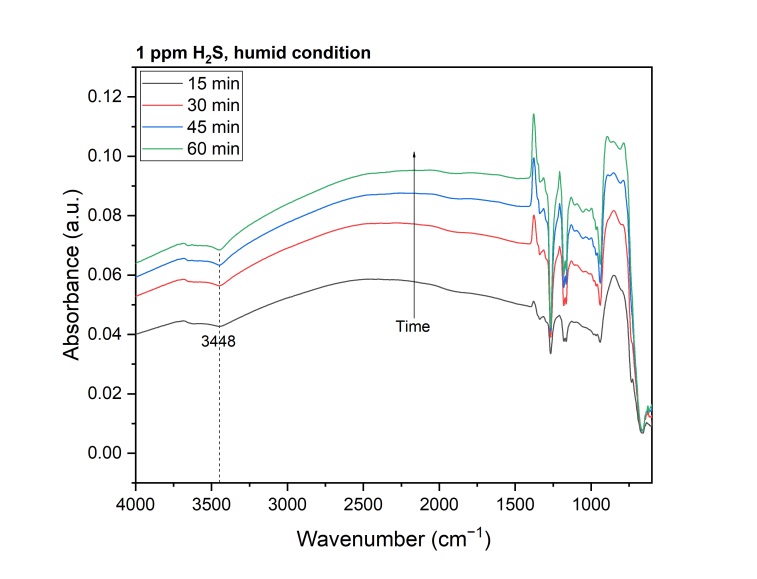


Figure S5 Absorbance spectra during the exposure to 1 ppm H_2_S at 300 °C in synthetic humid air in wavenumber range of 4000-600 cm^−1^. The selection of the corresponding reference and sample spectra are shown in Figure 6b.

**(a)**

**(b)**


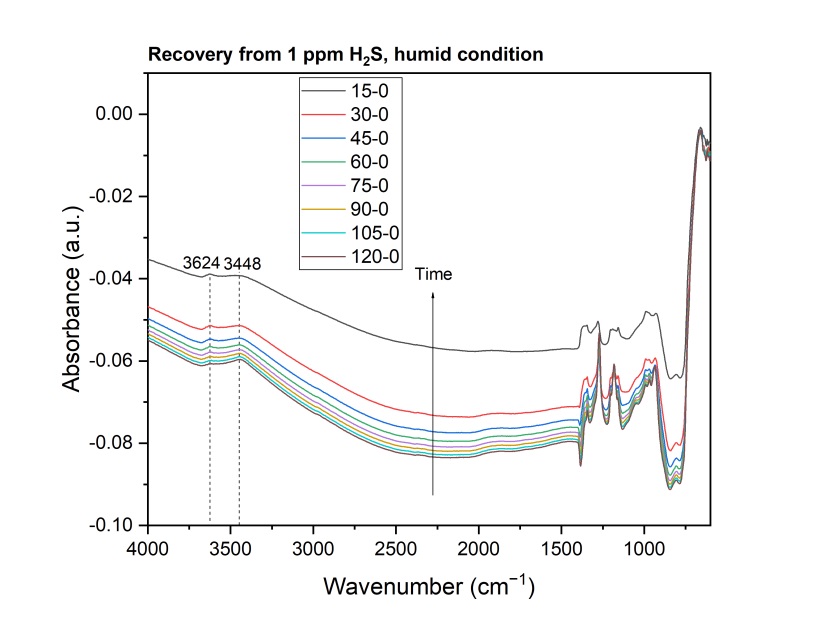

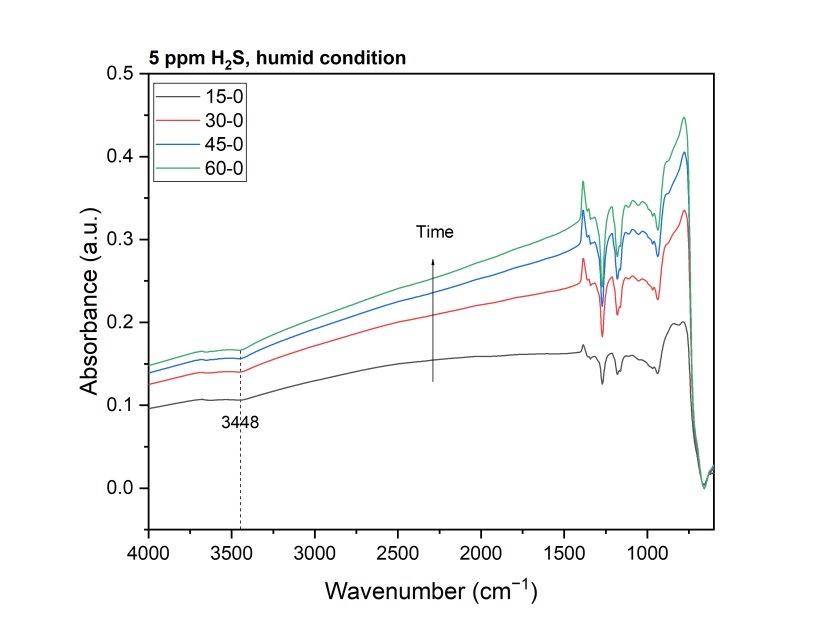


**(c)**


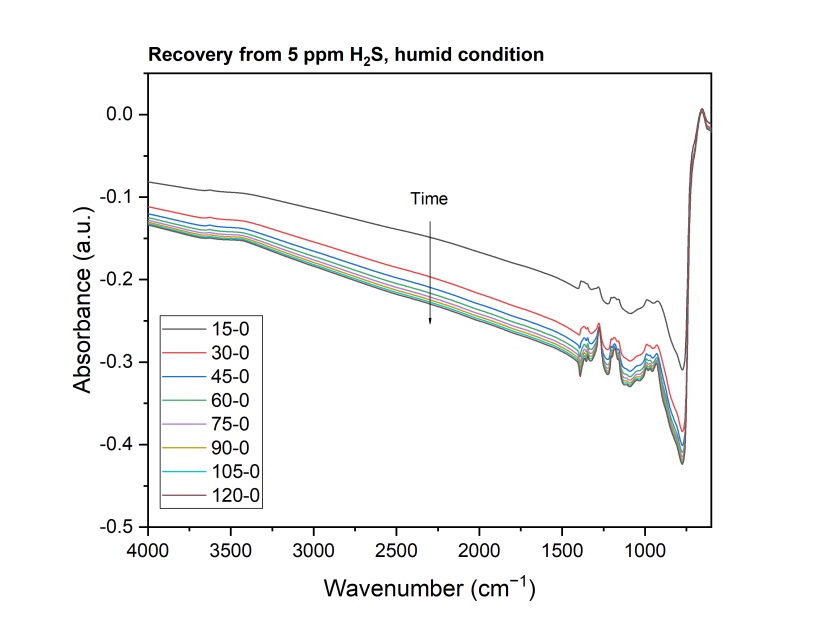


Figure S6 Absorbance spectra under humid condition: (a) during the recovery from 1 ppm H_2_S with referencing the spectra to the last SC spectrum in 1 ppm H_2_S, (b) during the exposure to 5 ppm H_2_S with referencing the spectra to the last SC spectrum during the recovery from 1 ppm H_2_S, and (c) during the recovery from 5 ppm H_2_S with referencing the spectra to the last SC spectrum in 5 ppm H_2_S. The selection of the corresponding reference and sample spectra are shown in Figure 7b, d, e.

**(b)**

**(a)**


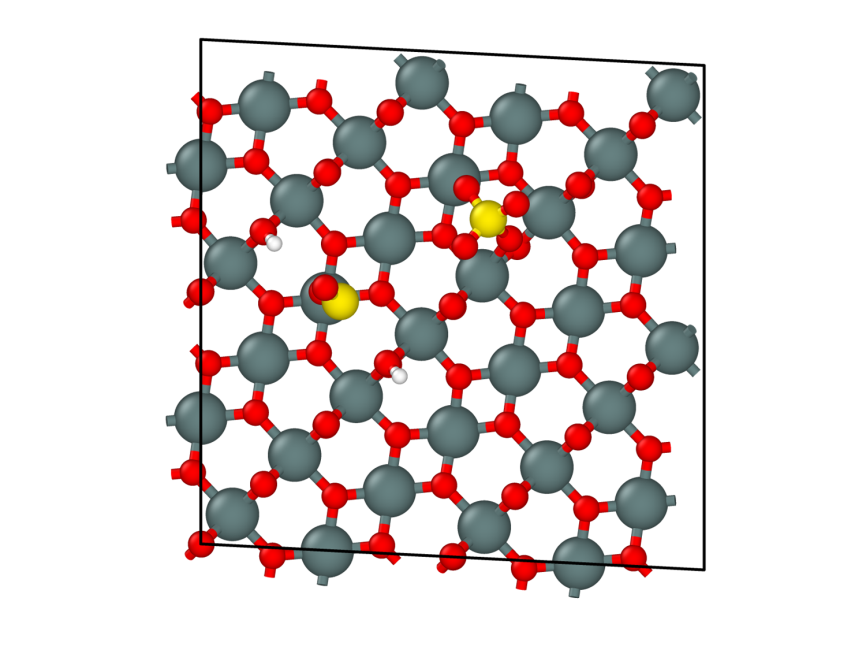

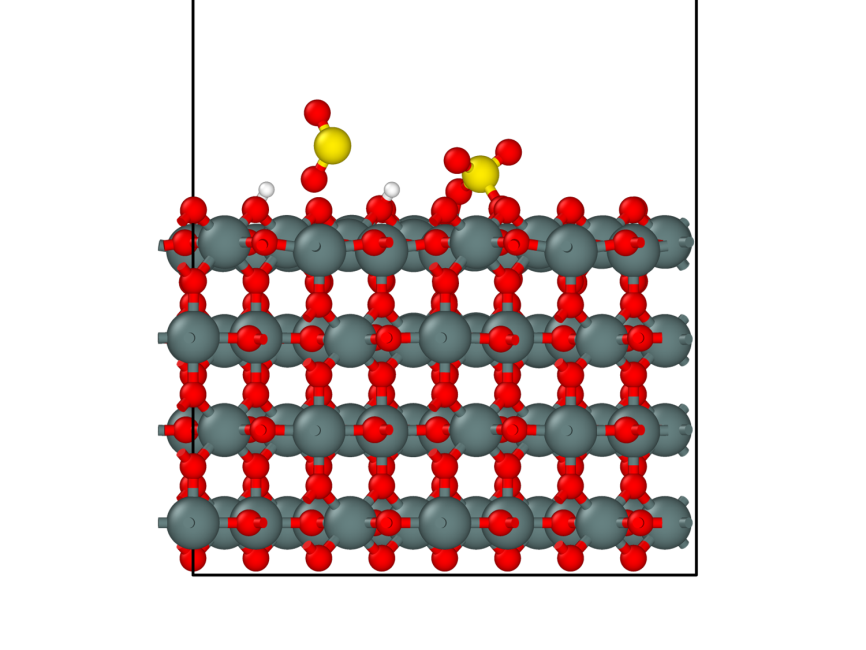


Figure S7 Adsorption configuration of SO_2_ oxidized from H_2_S at a SnO_2_ (110) surface with the presence of sulfate: (a) Top view and (b) side view.

**References**

[1] P. Giannozzi, S. Baroni, N. Bonini, M. Calandra, R. Car, C. Cavazzoni, D. Ceresoli, G. L. Chiarotti, M. Cococcioni, I. Dabo, A. Dal Corso, S. de Gironcoli, S. Fabris, G. Fratesi, R. Gebauer, U. Gerstmann, C. Gougoussis, A. Kokalj, M. Lazzeri, L. Martin-Samos, N. Marzari, F. Mauri, R. Mazzarello, S. Paolini, A. Pasquarello, L. Paulatto, C. Sbraccia, S. Scandolo, G. Sclauzero, A. P. Seitsonen, A. Smogunov, P. Umari, R. M. Wentzcovitch, *Journal of Physics: Condensed Matter* **2009**, *21*, 395502.

[2] https://www.quantum-espresso.org/pseudopotentials/.

[3] aA. Jay, C. Huet, N. Salles, M. Gunde, L. Martin-Samos, N. Richard, G. Landa, V. Goiffon, S. De Gironcoli, A. Hémeryck, N. Mousseau, *Journal of Chemical Theory and Computation* **2020**, *16*, 6726-6734; bA. Jay, M. Gunde, N. Salles, M. Poberžnik, L. Martin-Samos, N. Richard, S. d. Gironcoli, N. Mousseau, A. Hémeryck, *Computational Materials Science* **2022**, *209*, 111363.

[4] G. Henkelman, A. Arnaldsson, H. Jónsson, *Computational Materials Science* **2006**, *36*, 354-360.

[5] aY. Chen, X. Wang, C. Shi, L. Li, H. Qin, J. Hu, *Sensors and Actuators B: Chemical* **2015**, *220*, 279-287; bS. Wicker, M. Guiltat, U. Weimar, A. Hémeryck, N. Barsan, *The Journal of Physical Chemistry C* **2017**, *121*, 25064-25073; cX. Wang, H. Qin, Y. Chen, J. Hu, *The Journal of Physical Chemistry C* **2014**, *118*, 28548-28561; dZ. Lu, D. Ma, L. Yang, X. Wang, G. Xu, Z. Yang, *Physical chemistry chemical physics : PCCP* **2014**, *16*, 12488-12494.
